# Access to health services and its influence on adherence to treatment of arterial hypertension during the COVID-19 pandemic in a Hospital in Callao, Peru: A cross-sectional study

**DOI:** 10.12688/f1000research.141856.2

**Published:** 2024-04-15

**Authors:** Oriana Rivera-Lozada, Isabel Cristina Rivera-Lozada, Cesar Antonio Bonilla-Asalde

**Affiliations:** 1 South American Center for Education and Research in Public Health, Universidad Norbert Wiener, Lima, 15046, Peru; 2 Universidad del Cauca, Popayán, Cauca, Colombia; 3 Hospital Nacional Daniel Alcides Carrion, Callao District, Peru

**Keywords:** adherence; COVID-19; arterial hypertension; health services

## Abstract

**Background:**

Access to health services compromises therapeutic adherence in patients with arterial hypertension (HTN), which is a risk factor for cardiovascular disease and premature death. The aim of the research is to determine the influence of access to health services on adherence to antihypertensive treatment during the COVID-19 pandemic.

**Methods:**

We included a cross-sectional analytical study. A survey was applied to 241 hypertensive patients at the Daniel Alcides Carrión Hospital, Callao-Peru. Data were analyzed using SPSS software. Absolute and relative frequencies were reported and the chi-square test was applied with a statistical significance level of p<0.05. In addition, multiple logistic regression analysis was performed using the Stepwise method.

**Results:**

Our results show that non-adherence to treatment is associated with health expenses (ORa: 1.9 CI 95% 1.7-2.2), considers the environment clean (ORa: 1.4 IC 95% 1.2-1.8), not receiving care due to lack of a doctor (ORa: 2.8 CI 95% 1.5-3.2), difficult with procedures (ORa: 2.8 IC 95% 1.2-2.8), having difficulty with schedules (ORa: 3.7 CI 95% 2. 3-5.5), fear of receiving care at the hospital (ORa: 4.5 CI 95 % 2.7-6.8), trust in health staff (ORa: 7.5 CI 95% 2.3-10.5) and considering that the physician does not have enough knowledge (ORa: 3.1 CI 95% 2.4-7.8).

**Conclusion:**

Therapeutic adherence was associated with expenses in the consultation considers the environment clean, not receiving care due to lack of a doctor, difficult with procedures, having difficulty with schedules, fear of receiving care at the hospital, trust in health staff and considering that the physician does not have enough knowledge.

## Introduction

Non-communicable diseases (NCDs) are the cause of 71% (41 million) deaths per year worldwide, with cardiovascular diseases being the leading cause. ^1^ Arterial hypertension (HTN) is one of the main risk factors for the development of cardiovascular disease and is the leading cause of premature death worldwide, making it a threat to public health ^2^ despite the fact that early detection, treatment and adequate control make it possible to reduce its morbidity, mortality and complications. ^3^ This mainly affects middle- and low-income countries, complicating the achievement of the Sustainable Development Goals (SDGs), especially the reduction of premature deaths from NCDs by 33% by 2030. ^4^

In Peru, according to the Demographic and Family Health Survey 2020 -ENDES 2020 - the prevalence of arterial hypertension in the population aged 15 years and older is 16.4%, with notorious differences between males (21.3%) and females (12%), ^5^ where 68% of the population with the disease received treatment. ^6^ In this sense, hypertension control involves risk identification, appropriate treatment, lifestyle changes and adherence to treatment. ^7^

Adherence was defined according to the World Health Organization (WHO): “The degree to which a person’s behavior - taking medication, following a dietary regimen, and making lifestyle changes - corresponds to the agreed-upon recommendations of a health provider”. ^8^ Currently, adherence to treatment is an important point for the successful control of HTN. ^9^ However, Latin America registered heterogonous percentages associated mainly to low incomes and limitations to access to health care. ^10^ Guzman-Tordecilla *et al.* ^11^ reported medication adherence range between 46-94%. In the case of Peru, previous studies report that less than 50% of the population has optimal blood pressure levels and adherence to treatment. This situation makes them vulnerable to the development of complications such as acute myocardial infarction, stroke, among others. ^12^^–^^15^

In this sense, non-adherence to pharmacological treatment is a serious public health problem for the control of arterial hypertension, thus challenging health systems to improve their range of services. ^16^ In a current context of lessons learned from the COVID-19 pandemic, it is necessary to build services networks that answer to the needs of care and facilitate access to establishments with equity and quality, guaranteeing comprehensive health care in order to contribute to universal access of the population to establishments and that these act efficiently to face prevalent health problems, such as hypertension, thus protecting people’s lives. Health services must be actively involved and implement innovative strategies to ensure and effective and quick answer to recover the space lost during the COVID-19 pandemic. ^17^

Likewise, the control of hypertension can be affected by the limitations of health systems and the patient’s personal factors. ^7^ The literature mentions that access to health services can be explained thanks to facilitating elements and personal, geographic, economic, and health system barriers, among others. ^18^^,^^19^ Our paper considers Tanahashi’s definition of access to health care: “the interaction between specific aspects of service provision and the population that is influenced both by the characteristics of the health system and by the population’s resources and capacities to recognize needs and seek care”. ^9^

Due to the great global impact of HTN and its complications, in addition to the partial or total interruption in access and provision of health services for non-communicable diseases in the world caused by the COVID-19 pandemic, ^20^ it is more necessary than ever to ensure access to care for patients with HTN, since there has been a neglect of diseases other than those caused by the coronavirus from the beginning of 2020. ^21^ This is a challenge for weak health systems and for countries with limited resources, as it implies having policies that guarantee quality and equitable care that favors adherence to treatment and control of the disease. ^22^

Peru is a country with an economy in transition that is facing important structural challenges, as well as epidemiological, demographic, technological and health risk changes, leading to the increase of chronic noncommunicable diseases that emphasize health inequities and health impacts on vulnerable populations. ^23^ If we add the slowdown in health interventions to the situation described above as a result of the COVID-19 pandemic, all this raises challenges to the health system, where the approach to arterial hypertension and other chronic noncommunicable diseases becomes a sentinel indicator from the perspective of public health and the management of health services, becoming an expression of the good or bad that might result from the control strategies applied by the health system. ^24^

Even though several factors that contribute to nonadherence to antihypertensive treatment have been postulated, ^25^^–^^27^ it is important to develop studies to determine the influence of the determinants of access to health services on adherence to treatment, in order to prevent complications of the disease, in addition to having an impact on improving the Peruvian health system. Therefore, the aim of the present study was to determine the influence of access to health services on adherence to antihypertensive treatment during the COVID-19 pandemic.

## Methods

### Study design, instrument and population

A cross-sectional study was carried out between September-December 2021 in patients with a medical diagnosis of HTN who were seen in the cardiology and internal medicine clinics at the Daniel Alcides Carrion National Hospital in Peru. The inclusion criteria involved people over 18 years old, totally independent of their care, with at least 6 months of treatment, reading ability, and who would agree to sign the informed consent. Patients with metabolic comorbidities that require treatment, patients with complications of HTN, patients unable to answer the survey for medical reasons, and patients who did not prefer to participate in the study were excluded. The study took as reference a population of 1200 patients, an expected adherence to the therapeutic regime of 50%, 95% confidence interval, a design effect of 1 and losses due to non-response of 20%, resulting in a sample size of 291, 241 of which met the inclusion criteria. The sampling was systematic.

The survey included sociodemographic aspects and questions on access to health services according to the Tanahashi model, ^9^ which considers four stages in the access process to obtain quality coverage: availability, accessibility, acceptability and contact. Treatment adherence was measured using the Morisky-Green test, which included the following questions: 1. Do you ever forget to take your medication? 2. Are you careless at times about taking your medication? 3. When you feel better, do you sometimes stop taking your medication? 4. Sometimes, if you feel worse when you take the medication, do you stop taking it? We consider patients with HNT treatment adherence as those who answer the questions in the established order NO/YES/NO/NO. ^12^ The instruments were subjected to a reliability analysis, for which a pilot test was performed and a Cronbach’s Alpha of 0.88 was obtained for the Tanahashi model instrument, and a K-R of 0.85 for the Morisky-Green test.

### Procedures and statistical analysis

Data were collected in Excel 2010 format and analyzed using SPSS 21.0 software. Descriptive analysis included frequency distribution for sociodemographic, adherence variables, and dimensions of health care access. For the bivariate analysis, contingency tableswere performed and p-values were calculated using the chi-square test. The level of statistical significance was established with a value of p<0.05.

Below, a multiple logistic regression analysis was conducted using the Stepwise method. In this process, indicators with a p-value <0.05 in the bivariate analysis were progressively included, as well as variables with theoretical relevance that could have a significant impact on the model outcome due to their conceptual importance. ^28^

The regression equations were developed until no more indicators contributing to the model were found. The advantage of this method lies in the continuous evaluation of the predictors included in the model, so that the indicator explained by the remaining ones is eliminated.

### Ethical aspects

This study was conducted following the guidelines of the Declaration of Helsinki 1964 and its subsequent amendments. In addition, the study was approved by the Research Ethics Committee of the Hospital Daniel Alcides Carrion oficio N 2131 2021-HNDAC of the Callao region, Peru. All the participants in the study signed the informed consent form before their participation and their identity was anonymized for the elaboration of the database, so their integrity was not violated.

## Results

### Descriptive and bivariate analysis according to adherence to antihypertensive treatment in the study sample

A total of 241 adult patients with a diagnosis of hypertension were analyzed, of whom 65.15% (n=157) were female and 72.61% (n=175) were 60 years of age or older. In addition, 52.28% (n=126) were married or cohabitant. Likewise, 19.5% (n=47) had a university education. On the other hand, 21.99% (n=53) were not affiliated to the Public Health Insurance Scheme (SIS), 26.14% (n=63) indicated that they spent on medical consultations and 64.73% (n=156) spent on medicines. The bivariate analysis of the sociodemographic characteristics according to adherence is shown in Table 1.

**Table 1.** Descriptive and bivariate analysis according to adherence to antihypertensive treatment in adult patients with arterial hypertension treated at the Daniel Alcides Carrion National Hospital between September and December 2021.

Characteristics	n	%	Adherence to antihypertensive treatment		p-value ^1^
			Non-adherent (%)	Adherent (%)	
			n=224 (92.94)	n=17 (7.06)	
**Gender**					0.625
Female	157	65.15	145 (92.4)	12 (7.6)	
Male	84	34.85	79 (94.1)	5 (6.0)	
**Age**					0.117
168-37	9	3.73	9 (100.0)	-	
38-59	57	23.65	56 (98.3)	1 (1.8)	
≥ 60	175	72.61	159 (90.9)	16 (9.1)	
**Civil status**					0.285
Single	39	16.18	37 (94.9)	2 (5.1)	
Married or cohabitant	126	52.28	114 (90.5)	12 (9.5)	
Widowed or divorced	76	31.54	73 (96.1)	3 (4.0)	
**Schooling**					0.313
Never attended school	6	2.49	6 (100.0)	-	
Elementary school	49	20.33	48 (98.0)	1 (2.0)	
High school	139	57.26	125 (90.6)	13 (9.4)	
Higher education	47	19.50	44 (93.78)	3 (6.3)	
**Occupation**					0.571
Homemaker	144	59.75	132 (91.7)	12 (8.3)	
Student	4	1.66	4 (100.0)	-	
Laborer or tradesman	16	6.64	16 (100.0)	-	
Employee	13	5.39	13 (100.0)	-	
Unemployed	64	26.56	59 (92.2)	5 (7.8)	
**Employment status**					0.361
Pensioner	65	26.97	63 (96.9)	2 (3.1)	
Independent	57	23.65	52 (91.2)	5 (8.8)	
Dependent	9	3.73	9 (100.0)	-	
Unemployed	110	45.64	100 (90.9)	10 (9.1)	
**Family income**					0.004
≤ $750	73	30.29	71 (97.3)	2 (2.7)	
$751-1500	84	34.85	76 (90.5)	8 (9.5)	
>$1500	39	16.18	32 (82.1)	7 (18.0)	
Does not report	45	16.87	45 (100.0)	-	
**Public Health Insurance (SIS)**					0.824
Subsidy	183	75.93	171 (93.4)	12 (6.6)	
Semi-contributory subsidy	2	0.83	2 (100.0)	-	
Independent insurance	3	1.24	3 (100.0)	-	
Does not have SIS	53	21.99	48 (90.6)	5 (9.4)	
**Spends on consultations**					0.143
No	178	73.86	168 (94.4)	10 (5.6)	
Yes	63	26.14	56 (88.9)	7 (11.1)	
**Spends on medicines**					0.002
No	85	35.27	85 (100.0)	-	
Yes	156	64.73	139 (89.1)	17 (10.9)	
**Residence**					0.287
Family	98	40.66	93 (94.9)	5 (5.1)	
Rented	31	12.86	29 (93.6)	2 (6.5)	
Inherited	54	22.41	47 (87.0)	7 (13.0)	
Owned	58	24.07	55 (94.8)	3 (5.2)	

^1^ p-value estimated by chi-square test, with a significance level of p<0.05.

Regarding the characteristics and availability of health services, 66.8% (n=161) responded that they did not receive care because the physician was not available. In addition, when asked about the timely care received, 32.78% (n=79) reported not having received timely care for their consultations or examinations (29.88%, n=72). Finally, 46.89% (n=113) responded that they did not receive any information about their disease. **The bivariate analysis between the characteristics of the availability dimension of health services and adherence to hypertensive treatment revealed significant findings. It was found that maintaining a clean environment (p: 0.004), the availability of the doctor for care (p<0.001), and the preference for receiving care in the afternoon shift (p<0.001) were associated with higher adherence to hypertensive treatment.** The bivariate analysis of the characteristics and availability of health services according to adherence is shown in Table 2.

**Table 2.** Descriptive and bivariate analysis of the characteristics and availability of health services according to adherence to antihypertensive treatment in adult patients with arterial hypertension treated at the Daniel Alcides Carrion National Hospital between September and December 2021.

Characteristics	n	%	Adherence to antihypertensive treatment		p-value ^1^
			Non-adherent (%)	Adherent (%)	
			n=224 (92.94)	n=17 (7.06)	
**Considers the environment clean**					0.004
Does not know	1	0.41	1 (100.0)	-	
No	60	24.9	50 (83.3)	10 (16.7)	
Yes	180	74.69	173 (96.1)	7 (3.9)	
**Considers the bathrooms clean**					0.329
Does not know	11	4.56	9 (81.8)	2 (18.2)	
No	189	78.42	177 (93.7)	12 (6.4)	
Yes	41	17.01	38 (92.7)	3 (7.3)	
**When going to the physician's office, blood pressure is checked with a sphygmomanometer**					0.168
Does not know	4	1.66	4 (100.0)	-	
No	4	1.66	3 (75.0)	1 (25.0)	
Yes	200	82.99	184 (92.0)	16 (8.0)	
Not reported	33	13.69	33 (100.0)	-	
**Did not receive care because the physician was not available**					<0.001
Does not know	3	1.24	1 (33.3)	2 (66.7)	
No	161	66.8	154 (95.7)	7 (4.4)	
Yes	77	31.95	69 (89.6)	8 (10.4)	
**Would prefer to be seen in the afternoon shift**					<0.001
Does not know	4	1.66	4 (100.0)	-	
No	180	74.69	174 (96.8)	6 (3.3)	
Yes	57	23.65	46 (80.7)	11 (19.3)	
**Received timely attention for consultations**					0.193
No	79	32.78	71 (89.9)	8 (10.1)	
Yes	162	67.22	153 (94.4)	9 (5.6)	
**Received timely attention for exams**					0.184
Does not know	4	1.66	3 (75.0)	1 (25.0)	
No	72	29.88	65 (90.3)	7 (9.7)	
Yes	165	68.46	156 (94.6)	9 (5.5)	
**Knows the laboratory service**					0.024
Does not know	17	7.05	14 (82.4)	3 (17.7)	
No	55	22.82	55 (100.0)	-	
Yes	169	70.12	155 (91.7)	14 (8.3)	
**Received any information about the disease by a non-medical source ( broadcast media, family, and friends)**					0.200
Does not know	13	5.39	11 (84.6)	2 (15.4)	
No	113	46.89	103 (91.2)	10 (8.9)	
Yes	115	47.72	110 (95.7)	5 (4.4)	

^1^ p-value estimated by chi-square test, with a significance level of p<0.05.

Regarding accessibility, 24.48% (n=59) reported a travel time of more than 40 minutes, and the most frequently used means of transportation was the bus (78.42%, n=189). A total of 82.57% (n=199) responded that they considered that the health personnel were trained to provide care. Likewise, 58.51% (n=141) responded that they had had difficulties with administrative procedures, where the most common problem was the availability of the service (50.62%, n=122), while the least common was the lack of authorization of the service (0.41%, n=1).

On the other hand, it was found that 55.19% (n=133) considered inadequate the time they had to wait from the time they requested their appointment until they received care. Similarly, 49.8% (n=120) reported having waited more than 60 minutes to be seen on the day of their appointment and 39% (n=94) reported not having received timely care. Regarding the economic aspect, it was found that the median loss in soles for attending a consultation was 30.00 (RIQ=20). On the other hand, 33.2% (n=80) mentioned that they pay for the consultation or service received and 13.28% (n=32) have missed their appointment or care due to monetary limitations. Finally, 82.57% (n=199) bought medicines for their treatment and 46.47% (n=112) expressed not having complied with taking such medicine due to economic precariousness. The bivariate analysis of the characteristics of the accessibility dimension of health services and adherence to hypertensive treatment revealed several significant findings. It was found that experiencing difficulties with administrative procedures (p: 0.002), having difficulty in requesting care (p: 0.010), as well as difficulty in finding dates and times for care (p < 0.001), not considering waiting time adequate (p: 0.018), not receiving timely care (p: 0.009), or believing that attending the appointment is an economic loss (p: 0.042) were associated with lower adherence to hypertensive treatment. Table 3 shows the bivariate analysis of the accessibility of health services according to adherence.

**Table 3.** Descriptive and bivariate analysis of accessibility to health services according to adherence to antihypertensive treatment in adult patients with arterial hypertension treated at the Daniel Alcides Carrion National Hospital between September and December 2021.

Characteristics	n	%	Adherence to antihypertensive treatment		p-value ^1^
			Non-adherent (%)	Adherent (%)	
			n=224 (92.94)	n=17 (7.06)	
**Time delay from residence to hospital**					0.624
0-40 min	182	75.52	170 (93.4)	12 (6.6)	
>40 min	59	24.48	54 (91.5)	5 (8.5)	
**Means of transportation**					<0.001
Walking or cycling	14	5.81	10 (71.4)	4 (28.6)	
Taxi or motorcycle taxi	31	12.86	26 (83.9)	5 (16.1)	
Bus	189	78.42	182 (96.3)	7 (3.7)	
Own car	5	2.07	5 (100.0)	-	
Other	2	0.83	1 (50.0)	1 (50.0)	
**Care staff trained to provide care**					0.435
Does not know	22	9.13	19 (86.4)	3 (13.6)	
No	20	8.30	19 (95.0)	1 (50.0)	
Yes	199	82.57	186 (93.5)	13 (6.5)	
**Had difficulty with administrative procedures**					0.002
Not remember	1	0.41	1 (100.0)	-	
No	99	41.08	99 (100.0)	-	
Yes	141	58.51	124 (87.9)	17 (12.1)	
**Kind of difficulty in requesting care**					0.010
Difficulty with dates and times and availability of the service	90	37.34	78 (86.7)	12 (13.3)	
Lack of information, difficulties with dates, availability of the service	22	9.13	20 (90.9)	2 (9.1)	
None	88	36.5	88 (100.0)	-	
**Others (delayed appointments, lack of money to transport, and unkind hospital staff treatment)**	32	13.28	29 (90.6)	3 (9.4)	
No answer	9	3.73	9 (100.0)	-	
**Lack of information**					0.984
No	213	88.38	198 (93.0)	15 (7.0)	
Yes	28	11.62	26 (92.9)	2 (7.1)	
**Non-authorization of service**					0.783
No	240	99.59	223 (92.9)	17 (7.1)	
Yes	1	0.41	1 (100.0)	-	
**Difficulty of dates and times**					<0.001
No	116	48.13	115 (99.1)	1 (0.9)	
Yes	125	51.87	109 (87.2)	16 (12.8)	
**Personal financial aspects**					0.017
No	239	99.17	223 (93.3)	16 (6.7)	
Yes	2	0.83	1 (50.0)	1 (50.0)	
**Service availability**					0.001
No	119	49.38	117 (98.3)	2 (1.7)	
Yes	122	50.62	107 (87.7)	15 (12.3)	
**Additional procedures**					0.494
No	235	97.51	218 (92.8)	17 (7.2)	
Yes	6	2.49	6 (100.0)	-	
**Does not know**					0.783
No	240	99.59	223 (92.9)	17 (7.1)	
Yes	1	0.41	1 (100.0)	-	
**None**					0.001
No	153	63.49	136 (88.9)	17 (11.1)	
Yes	88	36.51	88 (100.0)	-	
**Other/which**					0.631
No	238	98.76	221 (92.9)	17 (7.1)	
Yes	3	1.24	3 (100.0)	-	
**They were last appointment was requested/**					0.072
1-3 days	112	46.47	108 (96.4)	4 (3.6)	
4-6 days	23	9.54	22 (95.7)	1 (4.4)	
7-9 days	106	43.98	94 (88.7)	12 (11.3)	
**Considers waiting time to be adequate**					0.018
Does not know	1	0.41	1 (100.0)	-	
No	133	55.19	118 (88.7)	15 (11.3)	
Yes	107	44.40	105 (98.1)	2 (1.9)	
**Waiting time on the day of appointment**					0.014
≤60 min	121	50.21	115 (95.0)	6 (5.0)	
61-180 min	72	29.88	69 (95.8)	3 (4.2)	
>180 min	48	19.92	40 (83.3)	8 (16.7)	
**Received timely care**					0.009
Does not know	7	2.90	5 (71.4)	2 (28.6)	
No	94	39.00	84 (89.4)	10 (10.6)	
Yes	140	58.09	135 (96.4)	5 (3.6)	
**Attending the consultation is a financial loss**					0.042
No	218	90.46	205 (94.0)	13 (6.0)	
Yes	23	9.54	19 (82.6)	4 (17.4)	
**How much is the economic loss estimated**	30 [20-40]	30 [20-40]	35 [25-55]		0.508
**A family member keeps company to the consultation**					0.343
No	191	79.25	176 (92.2)	15 (7.9)	
Yes	50	20.75	48 (96.0)	2 (4.0)	
**Amount lost by the person accompanying to the consultation**	30 [20-40]	27.5 [17.5-40]	45 [40-50]		0.130
**Any payment for consultation**					0.469
No	161	66.80	151 (93.8)	10 (6.2)	
Yes	80	33.20	73 (91.3)	7 (8.8)	
**Did not attend due to lack of money**					0.619
Does not know	1	0.41	1 (100.0)	-	
No	208	86.31	192 (92.3)	16 (7.79	
Yes	32	13.28	31 (96.9)	1 (3.1)	
**Purchased medicines for treatment**					0.049
No	42	17.43	42 (100.0)		
Yes	199	82.57	182 (91.5)		
**Did not take the medicines due to lack of money**					0.290
No	129	53.53	122 (94.6)		
Yes	112	46.47	102 (91.1)		
**Health services care have been denied in the last year**					<0.001
Does not know	28	11.62	21 (75.0)		
No	194	80.50	184 (94.9)		
Yes	19	7.88	19 (100.0)		

^1^ p-value estimated by chi-square test, with a significance level of p<0.05.

Regarding the acceptability of health services, it was found that 88.38% (n=213) reported that they did not feel afraid of being treated at the hospital and 96.68% (n=233) did not feel discriminated against or rejected because of their illness. Likewise, 93.78% (n=226) considered that the doctor had sufficient knowledge for their recovery, while 82.16% (n=198) trusted the health personnel in general. Finally, 16.6% (n=40) rated it as excellent, while 2.9% (n=7) rated the medical treatment as bad. The bivariate analysis of the characteristics within the acceptability dimension of health services and adherence to hypertensive treatment revealed significant findings. It was found that considering the physician to possess knowledge for recovery (p: 0.037), and having confidence in healthcare personnel (p: 0.012), were associated with adherence to hypertensive treatment. Table 4 shows the bivariate analysis of the acceptability of health services according to adherence.

**Table 4.** Descriptive and bivariate analysis of the acceptability of health services according to adherence to antihypertensive treatment in adult patients with arterial hypertension treated at the Daniel Alcides Carrion National Hospital between September and December 2021.

Characteristics	n	%	Adherence to antihypertensive treatment		p-value ^1^
			Non-adherent (%)	Adherent (%)	
			n=224 (92.94)	n=17 (7.06)	
**Fear of being treated at the hospital**					<0.001
Does not know	1	0.41	-	1 (100.0)	
No	213	88.38	202 (94.8)	11 (5.2)	
Yes	27	11.20	22 (81.5)	5 (18.5)	
**Felt discriminated against or rejected because of the disease**					0.428
No	233	96.68	216 (92.7)	17 (7.3)	
Yes	8	3.32	8 (100.0)	-	
**It is difficult for a neighbor or family member to know about one's health**					0.668
No	220	91.29	204 (92.7)	16 (7.3)	
Yes	21	8.71	20 (95.2)	1 (4.8)	
**Treatment will control hypertension**					0.646
Does not know	1	0.41	1 (100.0)	-	
No	10	4.15	10 (100.0)	-	
Yes	230	95.44	213 (92.6)	17 (7.4)	
**The physician will have sufficient knowledge for the recovery**					0.037
Does not know	2	0.83	1 (50.0)	1 (50.0)	
No	13	5.39	13 (100.0)	-	
Yes	226	93.78	210 (92.9)	16 (7.1)	
**Trusts in health staff**					0.012
Does not know	30	12.45	24 (80.0)	6 (20.0)	
No	13	5.39	12 (92.3)	1 (7.7)	
Yes	198	82.16	188 (95.0)	10 (5.1)	
**Feeling about the physician's treatment**					0.288
Bad	7	2.90	7 (100.0)	-	
Fair	20	8.30	17 (85.0)	3 (15.0)	
Good	174	72.20	161 (92.5)	13 (7.5)	
Excellent	40	16.60	39 (97.5)	1 (2.5)	

^1^ p-value estimated by chi-square test, with a significance level of p<0.05.

It was found that 34.02% (n=82) rated the quality of care as fair or very poor, while 5.81% (n=14) rated it as excellent or very good. Likewise, the quality of treatment received was perceived as fair or very poor by 8.71% (n=21) and as excellent by 27.39% (n=66) of the respondents. In addition, 70.95% (n=171) reported that the staff answered their questions, 95.85% (n=231) had their disease explained to them and 97.51% (n=235) received an explanation of the indicated treatment. It was found that 92.95% (n=224) were satisfied with the explanation provided by the health staff. In this study, no significant differences were found regarding the characteristics of the contact dimension and adherence to hypertensive treatment. Table 5 shows the bivariate analysis of the variables according to adherence.

**Table 5.** Descriptive and bivariate analysis of contact with health services according to adherence to antihypertensive treatment in adult patients with arterial hypertension treated at the Daniel Alcides Carrión National Hospital between September and December 2021.

Characteristics	n	%	Adherence to antihypertensive treatment		p-value ^1^
			Non-adherent (%)	Adherent (%)	
			n=224 (92.94)	n=17 (7.06)	
**Quality of care**					0.511
Fair or poor	82	34.02	75 (91.5)	7 (8.5)	
Good	145	60.17	135 (93.1)	10 (6.9)	
Excellent or very good	14	5.81	14 (100.0)	-	
**Quality of treatment received**					0.320
Fair or poor	21	8.71	19 (90.5)	2 (9.5)	
Good	154	63.90	146 (94.8)	8 (5.2)	
Excellent or very good	66	27.39	59 (89.4)	7 (10.6)	
**Staff answered the concerns**					0.572
Does not know	55	22.82	52 (94.6)	3 (5.5)	
No	15	6.22	13 (86.7)	2 (13.3)	
Yes	171	70.95	159 (93.0)	12 (7.0)	
**Had any discomfort with the medication**					0.598
No	186	77.18	172 (93.6)	14 (7.5)	
Yes	55	22.82	52 (94.6)	3 (5.5)	
**Stopped taking the medication before completing treatment**					0.444
No	188	78.01	176 (93.6)	12 (6.4)	
Yes	53	21.99	48 (90.6)	5 (9.4)	
**Health staff explained about the disease**					0.374
No	10	4.15	10 (100.0)	-	
Yes	231	95.85	214 (92.6)	17 (7.4)	
**Health staff explained about the treatment**					0.494
No	6	2.49	6 (100.0)	-	
Yes	235	97.51	218 (92.8)	17 (7.2)	
**Satisfied with the explanation**					0.500
Does not know	7	2.90	7 (100.0)	-	
No	10	4.15	10 (100.0)	-	
Yes	224	92.95	207 (92.4)	17 (7.6)	

^1^ p-value estimated by chi-square test, with a significance level of p<0.05.

Multiple logistic regression analysis according to adherence to antihypertensive treatment in the study sample.

The multiple logistic regression model found an association between adherence to antihypertensive treatment and expenses in the consultation (ORa: 1.9, CI 95% 1,7:2,2), considers the environment clean (ORa: 1.4 IC 95% 1.2-1.8), not receiving care due to lack of a doctor (ORa: 2.8 CI 95% 1.5-3.2), difficult with procedures (ORa: 2.8 IC 95% 1.2-2.8), having difficulty with schedules (ORa: 3.7 CI 95% 2.3-5.5), fear of receiving care at the hospital (ORa: 4.5 CI 95% 2.7-6.8), trust in health staff (ORa: 7.5 CI 95% 2.3-10.5) and considering that the physician does not have enough knowledge (ORa: 3.1 CI 95% 2.4-7.8). Table 6 shows all the associations found.

**Table 6.** Logistic regression analysis according to adherence in adult patients with arterial hypertension seen at the Daniel Alcides Carrión National Hospital between September and December 2021.

Characteristics	Adjusted OR		CI 95%	p-value ^1^
Expenses in the consultation	No	1.9	1.7 – 2.2	<0.001
	Yes			
Considers the environment clean	No	1.4	1.2 – 1.8	0.001
	Yes			
Not receiving care because the physician was not present	No	2.8	1.5 – 3.2	<0.001
	Yes			
Difficulty with procedures	No	1.8	1.2 – 2.8	0.035
	Yes			
Difficulty with dates and schedules	No	3.7	2.3 – 5.5	0.040
	Yes			
Fear of being treated at the hospital	No	4.5	2.7 – 6.8	<0.001
	Yes			
Trusts in health staff	No	7.5	2.3 – 10.5	<0.001
	Yes			
Considers that the physician did not have enough knowledge to treat	No	3.1	2.4 – 7.8	0.030
	Yes			

^1^ p-value estimated by chi-square test, with a significance level of p<0.05.

## Discussion

In this study, non-adherence to antihypertensive treatment reached 92.94%, this predominance of non-adherence to treatment in hypertensive patients was also reported in a study on therapeutic adherence in patients with chronic diseases, where Bertoldo *et al.* ^29^ found that 38% of patients did not comply with treatment, highlighting hypertensive patients with 75% non-adherence. However, this high percentage found does not coincide with the general acceptance of therapeutic adherence which is between 50 and 70% and differs from the 37.4% adherence reported by Quintana *et al.* ^30^ or the 43.9% reported by Martinez ^31^ using, in both cases, the same instrument.

Likewise, it differs from Peruvian studies where the Morisky-Green test was also used, such as that of Carhuallanqui *et al.* ^32^ who found 37.9% adherence and that of Fernández-Arias ^14^ who found 57.4% adherence in hypertensive patients. However, the low adherence rate coincides with that found by Rosas-Chávez ^33^ in an observational study, where they determined a 15% adherence rate, although it is still above the percentage found in the present study, this low adherence is a sample of the heterogeneity of this phenomenon in Peru, probably related to cultural, demographic and educational factors, considering that both studies were carried out in different hospitals in Lima and Callao.

On the other hand, in the United States, lack of adherence to antihypertensive treatment affects approximately 75% of patients, which implies that they do not achieve optimal blood pressure control. In addition, studies carried out in recent years show that about 50% of hypertensive patients are unable to comply with a hygienic-health regimen and to adhere correctly to pharmacological treatment, especially when these measures last for more than 1 year. ^34^

Likewise, the evidence reviewed suggests that sociodemographic characteristics such as gender and age, among others, seem to be related to adherence. ^25^ This does not coincide with the findings of this study, since none of the sociodemographic characteristics studied was significantly associated with adherence, with the exception of family income (p=0.004), where the results showed a greater number of adherent patients (9.5%) among those with an income of between 751 and 1500 dollars. Despite the above, the percentage remains low; and expenditure on medications (p=0.002) where 100% non-adherence was found among those who do not spend on medications, probably due to the fact that those who spend on their health tend to take better care of themselves. The findings of Ruiz-Alejos *et al.* ^26^ and Martínez *et al.* ^26^ show that arterial hypertension predominates in the male gender, in contrast to this study, which found a predominance in the female sex (65.15%).

On the other hand, in order to study the reasons for this low adherence to antihypertensive treatment, Tanahashi suggested the need to focus attention on access to health services to identify the population that does not have access or has difficulty in doing so and to redirect actions towards them to improve primary care coverage. ^35^ This is all the more important because of the asymptomatic nature of hypertension and, therefore, in many cases it is detected as a finding in a routine examination and most patients are unaware that they suffer from hypertension, ^36^ so early detection of hypertension is crucial in Peru and relies mainly on routine blood pressure control in patients who come for consultation. However, this is not consistent with what was found in this study, because although 82.99% of patients had their blood pressure checked at the time of consultation, it was not found that receiving such control was significantly associated with adherence (p=0.168). It also differs from what was reported by Gabert *et al.,* ^27^ in that study, which also refers to the scarcity or deterioration of resources and personnel to carry out an adequate diagnosis, as in the present study, since the unavailability of the physician to provide care (p<0.001), as well as the availability of the services (p=0.001), not knowing the location of some services such as the laboratory (p=0.024) and the hygienic state of the environments where care was received (p=0.004) were associated with therapeutic adherence.

In addition, adherence may also be compromised by the patient’s confidence in receiving care and his or her relationship with the health care provider, ^37^ demonstrating that fear of receiving care in the hospital is associated with adherence (p<0.001).

It is important to mention the importance of patient follow-up after the first visit to control the progression of the disease and reduce the possible risk factors that the patient presents, taking into account the cardiovascular risk, which should also be evaluated at the first visit. Carrying out the aforementioned can be complicated by the availability of patients who work or fulfill obligations that demand a large part of their day, ^38^ this coincides with what was found in this study, since the timetable difficulty was associated with adherence (p<0.001) as well as the waiting time to receive an appointment (p=0.018) and to be seen on the scheduled day (p=0.014).

Other authors, such as Gabert ^27^ and Owolabi ^39^ raised the availability and accessibility of adequate medications as important barriers to the management of hypertension, similar to what has been found in other studies on access to treatment in other chronic diseases, all of which coincides with the results of this study that identified as factors associated with adherence the means of transportation (p<0.001) and difficulty with administrative procedures (p=0.002). As consequence, in this study, not having received timely care was related to adherence (p=0.009).

Likewise, the family plays a fundamental role in compliance with the therapeutic regimen, providing support in seeking care and, in many cases, assuming a leadership role during treatment. ^30^ However, no significant association was found between therapeutic adherence and family or acquaintance support (p=0.428), This could be explained by the lack of specificity of the question in the Morisky-Green test, since it does not have a question directed to this point and the closest questions are marital status and a question related to the difficulty of a family member or neighbor knowing about the patient’s health, which leaves little or no information available to study this possible relationship.

The limitations of the present study include: First, the methodologic design of our study does not allow us to estimate a causal relationship. Second, we used a nonprobabilistic sampling method. This limits generalizing the conclusion because our sample could not be representative. Finally, our results cannot be extrapolated to different populations.

On the other hand, a systematic review conducted by AlGhurair *et al.* ^40^ found a consistent and significant association between economic costs and non-adherence to antihypertensive treatment. This finding reflects that healthcare expenses are strongly linked to a higher likelihood of non-adherence. Such association suggests the necessity of healthcare policies addressing the economic costs related to treatment, especially in pandemic contexts where financial difficulties may worsen.

Additionally, studies such as that of Wei *et al.* ^41^ Have emphasized the importance of trust in healthcare personnel and the perceived quality of medical care in adherence to treatment for chronic diseases. Our finding that trust in healthcare personnel is positively associated with adherence reinforces this notion. Trust in healthcare providers can influence patients’ willingness to follow treatment recommendations, underscoring the importance of building trustful relationships between patients and healthcare professionals.

Furthermore, our study also identified additional factors that may affect treatment adherence, such as the perception of a clean environment, difficulties with medical procedures, and inadequate appointment scheduling. These findings align with previous research highlighting the significance of comfort and accessibility of healthcare services in treatment adherence. ^42^^,^^43^

The implications of our findings are significant for clinical practice and public health policies. Firstly, our results emphasize the need for interventions addressing financial barriers to access antihypertensive treatment, especially during crises like the COVID-19 pandemic. This may involve cost-reduction strategies such as subsidized health insurance programs or free access to essential medications, alongside promoting safe healthcare environments and fostering trustful communication between doctors and patients.

Regarding the study limitations, we acknowledge that the cross-sectional design used does not allow for establishing causal relationships, and the non-probabilistic sampling method may limit the generalizability of our findings. Nevertheless, these results offer valuable insights into the challenges faced by hypertensive patients during the COVID-19 pandemic and underscore the necessity of effective, patient-centered interventions to enhance treatment adherence. In summary, our findings contribute to the body of knowledge on antihypertensive treatment adherence and provide a solid foundation for developing interventions aimed at improving clinical outcomes and reducing disease burden among hypertensive patients during the COVID-19 pandemic.

## Conclusions

The adherence to antihypertensive treatment evaluated in our study sample is associated with family income, medicine expenses, availability of the physician for care, means of transportation, difficulty of dates and schedules, fear of being treated in the hospital, trust in health staff, availability of services, waiting time, and receiving timely care.

The adherence to antihypertensive treatment evaluated in our study sample is associated with expenses in the consultation considers the environment clean, not receiving care due to lack of a doctor, difficult with procedures, having difficulty with schedules, fear of receiving care at the hospital, trust in health staff and considering that the physician does not have enough knowledge.

In conclusion, our study contributes to the growing body of evidence emphasizing the importance of access to healthcare services in adherence to antihypertensive treatment. Individual and systemic interventions are needed to address the identified barriers and improve treatment adherence, especially in times of public health crises such as the COVID-19 pandemic.

## Data Availability

Zenodo: Access to health services and its influence on adherence to treatment of arterial hypertension during the COVID-19 pan-demic in a Hospital in Callao, Peru: A Cross-Sectional Study, https://doi.org/10.5281/zenodo.8299902. ^44^ This project contains the following underlying data:
• DATABASE_.xlsx (dataset)

## Extended data

Zenodo: Access to health services and its influence on adherence to treatment of arterial hypertension during the COVID-19 pan-demic in a Hospital in Callao, Peru: A Cross-Sectional Study, https://doi.org/10.5281/zenodo.8299902. ^44^

This project contains the following extended data:

• Survey (a copy of the questionnaire)

Data are available under the terms of the Creative Commons Attribution 4.0 International license (CC-BY 4.0).
