# Supplementary material for: Access to health services and its influence on adherence to treatment of arterial hypertension during the COVID-19 pandemic in a Hospital in Callao, Peru: A cross-sectional study
Source: F1000Res. 2024 Apr 15;12:1215. Originally published 2023 Sep 26. [Version 2] doi: 10.12688/f1000research.141856.2 (PMC11043659; doi:10.12688/f1000research.141856.2)
Supplement: Peer review history

# Reviewer report: Reviewer response for version 2

**DOI:** 10.5256/f1000research.164150.r266495

**Published:** 2024-04-26

**Authors:** Hans Contreras Pulache

**Affiliations:** 1 Investigación y Desarrollo, EDUNEURO, Peru, Peru

There is no comments to this new version.

Is the work clearly and accurately presented and does it cite the current literature?

Yes

If applicable, is the statistical analysis and its interpretation appropriate?

Yes

Are all the source data underlying the results available to ensure full reproducibility?

Yes

Is the study design appropriate and is the work technically sound?

Yes

Are the conclusions drawn adequately supported by the results?

Yes

Are sufficient details of methods and analysis provided to allow replication by others?

Yes

Reviewer Expertise:

Public health, epidemiology.

I confirm that I have read this submission and believe that I have an appropriate level of expertise to confirm that it is of an acceptable scientific standard.

# Reviewer report: Reviewer response for version 2

**DOI:** 10.5256/f1000research.164150.r266496

**Published:** 2024-04-24

**Authors:** Salomon Huancahuire-Vega

**Affiliations:** 1 Peruvian Union University UPeU, Lima, Peru

criticisms were corrected

Is the work clearly and accurately presented and does it cite the current literature?

Yes

If applicable, is the statistical analysis and its interpretation appropriate?

Yes

Are all the source data underlying the results available to ensure full reproducibility?

Yes

Is the study design appropriate and is the work technically sound?

Yes

Are the conclusions drawn adequately supported by the results?

Partly

Are sufficient details of methods and analysis provided to allow replication by others?

Yes

Reviewer Expertise:

Public health

I confirm that I have read this submission and believe that I have an appropriate level of expertise to confirm that it is of an acceptable scientific standard.

# Reviewer report: Reviewer response for version 1

**DOI:** 10.5256/f1000research.155340.r210110

**Published:** 2024-02-22

**Authors:** Salomon Huancahuire-Vega

**Affiliations:** 1 Peruvian Union University UPeU, Lima, Peru

This is an interesting work on the influence of access to health services on adherence to antihypertensive treatment during the COVID-19 pandemic in a Peruvian hospital. This work is well planned, uses good instruments to measure the variables, and reports important data to take into consideration so that patients can have better adherence to treatment. However, there are some aspects of the manuscript that remain unclear and imprecise, and that must be clarified and corrected:

 1. In the summary, results section, 6 characteristics associated with adherence are mentioned. It is understood from the description that these associations were obtained from the regression analysis; however, in Table 6 (logistic regression) 8 associations are mentioned. Why not mention the two missing ones? (considers the environment clean, difficulty with procedures)

 2. Similarly, in the results section (multiple logistic regression analysis) (page 10) only three associations are mentioned.

 3. In the conclusion of the abstract and the manuscript, waiting time is mentioned as an element associated with treatment adherence, however, this result is not shown in the regression analysis.

 4. In the methodology section, according to the calculation of the sample size mentioned, this was not achieved. Why was this not achieved? This should be mentioned as a limitation of the study.

 5. The description of the dimensions of the instrument used to measure access to health services indicates: availability, accessibility, acceptability, contact, and effective coverage. However, there are no results for the last dimension.

 6. In the results section it is mentioned that 4.5% of participants had a university education, however, table 1 indicates 57.26%

 7. The report of results in tables 2 – 5 focuses on percentages according to the responses to each question of the instruments. It would be more interesting and the information in these tables would be better utilized if their description focused on the significance obtained from the bivariate analysis.

 8. Why was the characteristic “expenses in the consultation” included in the multiple regression analysis if this characteristic did not show a significant association in the bivariate analysis (table 1)?

 9. The data in Table 6 show the adjusted OR values. It would be interesting to show the raw ORs and mention which characteristics were considered to make the adjusted analysis.

 10. The discussion section should focus in more detail on those elements or characteristics that were shown to have an influence on treatment adherence (from the regression analysis), rather than the prevalence of treatment adherence.

 11. It would be interesting to add to the discussion what the implications of the results of this study would be, as well as mention other limitations of the study. For example, since the study was done during a pandemic, it was important to have information on how many of the participants had the infection.

 12. The conclusion of the work mentions “family income”, “means of transportation” and “waiting time” as elements associated with adherence, however, this is not reported in the results section. These inaccuracies must be corrected in the different sections of the manuscript.

Is the work clearly and accurately presented and does it cite the current literature?

Yes

If applicable, is the statistical analysis and its interpretation appropriate?

Yes

Are all the source data underlying the results available to ensure full reproducibility?

Yes

Is the study design appropriate and is the work technically sound?

Yes

Are the conclusions drawn adequately supported by the results?

Partly

Are sufficient details of methods and analysis provided to allow replication by others?

Yes

Reviewer Expertise:

public health

I confirm that I have read this submission and believe that I have an appropriate level of expertise to confirm that it is of an acceptable scientific standard, however I have significant reservations, as outlined above.

# Reviewer report: Reviewer response for version 1

**DOI:** 10.5256/f1000research.155340.r210111

**Published:** 2023-11-17

**Authors:** Hans Contreras Pulache

**Affiliations:** 1 Investigación y Desarrollo, EDUNEURO, Peru, Peru

I consider this work to be adequately supported, with a noteworthy level of methodological detail. Furthermore, it is likely that this study will become a fundamental reference for education and research in Peru (given its original focus on the subject matter).

 To further enrich the discussion, I suggest adding a paragraph in the discussion section that alludes to the low prevalence of treatment adherence (less than 10%) and the implications this has for future designs, specifically what parameters would be recommended for consideration regarding sample size calculations.

Is the work clearly and accurately presented and does it cite the current literature?

Yes

If applicable, is the statistical analysis and its interpretation appropriate?

Yes

Are all the source data underlying the results available to ensure full reproducibility?

Yes

Is the study design appropriate and is the work technically sound?

Yes

Are the conclusions drawn adequately supported by the results?

Yes

Are sufficient details of methods and analysis provided to allow replication by others?

Yes

Reviewer Expertise:

Public health, epidemiology.

I confirm that I have read this submission and believe that I have an appropriate level of expertise to confirm that it is of an acceptable scientific standard.
